# Physicians’ and patients’ perceived risks of chronic pain medication and co-medications in Quebec, Canada: a cross-sectional study

**DOI:** 10.1186/s12875-025-02704-5

**Published:** 2025-01-14

**Authors:** Gwenaëlle De Clifford-Faugère, Anaïs Lacasse, Hermine Lore Nguena Nguefack, Marimée Godbout-Parent, Aline Boulanger, Nancy Julien

**Affiliations:** 1 Département des sciences de la santé, Université du Québec en Abitibi-Témiscamingue (UQAT), Rouyn-Noranda, Québec Canada; 2 Centre d’expertise en gestion de la douleur chronique, Centre Hospitalier de l’Université de Montréal, Université de Montréal, Montréal, QC Canada; 3 Département d’anesthésiologie et de médecine de la douleur, Faculté de médecine, Université de Montréal, Montréal, Québec Canada

**Keywords:** Chronic pain, Medication quantification scale (MQS), Physician patient relationship, Medication risks, Patient education, Adverse drug reaction

## Abstract

**Background:**

The risks associated with medications and co-medications for chronic pain (CP) can influence a physician’s choice of drugs and dosages, as well as a patient’s adherence to the medication. High-quality care requires patients to participate in medication decisions. This study aimed to compare perceived risks of medications and co-medications between physicians and persons living with CP.

**Methods:**

This cross-sectional survey conducted in Quebec, Canada, included 83 physicians (snowball sampling) and 141 persons living with CP (convenience sampling). Perceived risks of adverse drug reaction of pain medications and co-medications were assessed using 0–10 numerical scales (0 = no risk, 10 = very high risk). An arbitrary cutoff point of 2-points was used to ease the interpretation of our data. Physicians scored the 36 medication subclasses of the Medication Quantification Scale 4.0 (MQS 4.0) through an online survey, while CP patients scored the medication subclasses they had taken in the last three months through telephone interviews.

**Results:**

Persons living with CP consistently perceived lower risks of adverse drug reaction compared to physicians. For eight subclasses, the difference in the mean perceived risk score was > 2 points and statistically significant (*p* < 0.05): non-specific oral NSAIDs, acetaminophen in combination with an opioid, short-acting opioids, long-acting opioids, tricyclic antidepressants, antipsychotics, benzodiazepines, and medical cannabis.

**Conclusions:**

Divergent risk perceptions between physicians and patients underscore the necessity of facilitating a more extensive discussion on medications and co-medications risks to empower patients to make informed decisions and participate in shared decision-making regarding their treatments.

**Supplementary Information:**

The online version contains supplementary material available at 10.1186/s12875-025-02704-5.

## Background

Chronic pain (CP), defined as pain persisting or recurring beyond three months [1], affects over 20% of Canadians [2] and can have significant physical, psychological, and social consequences [3]. Persons with CP often experience sleep disturbances, limitations in daily activities, decreased quality of life, social withdrawal, and mental health issues, including a high risk of depression and suicidal ideation [3].

A multimodal approach combining physical, psychological, and pharmacological therapies is recommended for CP treatment [4]. Physical and psychological therapies are considered in first-line therapies [5], but persons with CP are often prescribed various analgesics and coanalgesics, such as anti-inflammatory drugs, opioids, antidepressants, and anticonvulsants [6]. However, these medications can lead to adverse drug reactions [6, 7], which vary depending on the medication, duration of use, number of medications used, and individual characteristics [6–8]. An adverse drug reaction is defined as a response to a drug which is noxious and unintended, and which occurs at doses normally used for prophylaxis, diagnosis, or therapy of disease or the modification of physiologic function [9, 10]. Recent evidence has shown that among persons living with CP, the frequency of polypharmacy (using ≥ 5 medications) is very high (71%) [11]. Even if polypharmacy can be rational, persons living with CP have a greater likelihood of living with at least 3 chronic diseases [12], increasing their chances of using more medications, and consequently being exposed to more adverse drug reactions and medication-related problems [13].

The perception of risks associated with medication can vary between physicians and persons living with CP. Physicians possess in-depth knowledge of pathologies, pharmacology, evidence about adverse drug reactions, and risks of medication interactions, combined with their clinical experience. They are particularly vigilant about serious adverse drug reactions and dependence-prone medications, such as opioids [14]. On the other hand, persons living with CP may have experienced different medications and know what works for them in terms of type, dosage, and timing. Adverse drug reactions caused by medication may lead to the need to avoid or reduce the dose of certain medications [15–18] to preserve their quality of life, even if its impact pain relief.

To facilitate the therapeutic alliance between physician and patient, it is important that they work together in a shared decision-making perspective, discussing potential differences in the perceived risks associated with medication to achieve optimal therapy for the person living with CP. This study aimed to describe and compare physicians’ and persons living with CP’s perceptions of the risks of adverse drug reactions for the different medications and co-medications used for CP and associated comorbidities (e.g., sleep disorders, depression, anxiety). Physicians are relying on clinical experience and evidence-based practices to assess risks, while patients draw on personal medication experiences and lay knowledge. Although these two stakeholder groups certainly approach medications’ risks from distinct perspectives, examining these contrasting viewpoints may reveal significant gaps warranting further attention.

## Methods

The present study is part of a larger initiative on perceptions of the risks associated with the use of medications among persons living with CP (analgesics and co-medications) [12]. Ethical approval was obtained from the Research Ethics Board of Université du Québec in Abitibi-Témiscamingue (#2020-01–Diallo, M.A). Informed consent to participate was obtained from all participants. This manuscript was written in accordance with the STrengthening the Reporting of OBservational Studies in Epidemiology (STROBE) statement [19].

### Study design and setting

A cross-sectional study was conducted from February to June 2022 in Quebec, Canada. Snowball sampling was used to recruit physicians. To be eligible, physicians had to: (1) report dispensing and/or adjusting prescriptions for the treatment of CP in their clinical practice, (2) hold a valid license and practice in a Canadian setting, and (3) be able to complete a questionnaire in French. Persons living with CP were recruited through a convenience sample and had to: (1) report having pain persisting or recurring for more than 3 months, regardless of the cause, (2) have used medication for pain management in the past year, (3) be over 18 years of age, and (4) be able to complete a telephone interview in French. Telephone interviews were conducted for patients, as pharmacotherapy is complex and may require guidance to properly categorize the information.

### Procedure

#### Physicians’ recruitment

Physicians were recruited through web platforms held by Quebec professional associations and research networks (social networks, associations’ and networks’ newsletters), as well as through email sent by the team members (“snowball” sampling). The web link (URL) to access the anonymous online questionnaire on the SurveyMonkey^®^ platform was provided in the study invitation (voluntary survey). After confirming that they had read the consent form and consented to the research, physicians accessed the eligibility requirements (boxes to check) and then the questionnaire. *Persons living with CP recruitment*. In a previous project conducted by AL involving the recruitment of nearly 2000 persons living with CP in Quebec (ChrOnic Pain trEatment [COPE] Cohort) [20], participants were asked if they would be willing to be recontacted by email to participate in future studies. The COPE Cohort participants had originally been recruited from across all Quebec regions (*n* = 17) and were shown to be representative of random samples of Canadians with chronic pain in terms of age, employment status, level of education, pain duration, pain intensity, and most common pain locations [20]. For this study, an email invitation containing a project presentation, information, and consent form were sent to those who had agreed to be recontacted. Individuals willing to participate were invited to contact us by email. Responding to the email and providing availability for an interview constituted written consent. Invitations were sent until a sample of at least 140 individuals was reached, allowing for representation of users from the main classes of medications used in CP (analgesics and co-medications). Three adequately trained research assistants conducted telephone interviews, and meetings were held to ensure consistency in the questionnaire administration. Responses were manually entered into the computerized questionnaire version on the SurveyMonkey^®^ platform.

### Measures

Both the physicians’ and patients’ questionnaires covered the 36 medication subclasses of the validated Medication Quantification Scale 4.0 (MQS-4.0) [12], which includes a list of medications (analgesics and co-medications) commonly used by persons living with CP for pain management and associated comorbidities (e.g., sleep, mood). Medical cannabis is also listed. The items of the MQS-4.0 [12], which were presented to physicians and patients, are presented in Additional file 1. Since the MQS [12, 21] encompasses a broader range than just analgesic medication (e.g., all types of antidepressants, antipsychotics, corticosteroids, clonidine, barbiturates), it was expected that some subclasses would be less frequently used in our sample.

#### Questionnaire for physicians

The self-reported anonymous web-based questionnaire took approximately 20 min to complete and included 12 questions about sociodemographic (sex at birth, gender identity, region of residence) and practice profiles. Participants were also asked to assign a score between 0 and 10 reflecting their perception of the overall risk associated with each medication subclass of the MQS-4.0 (0 representing no risk and 10 a very high risk). The definition of the overall risk of medication was specified to participants before survey completion, i.e., the risk of medication causing short- or long-term adverse drug reactions, such as organ-specific or systemic toxicity (gastrointestinal symptoms, central nervous system), medication interactions, physical/psychological dependence potential, abuse potential, insomnia, tolerance, increased pain perception over time (hyperalgesia), and memory or concentration problems [12]. Examples of various medications in each subclass were presented. The questionnaire was pre-tested with 11 individuals (these data were excluded from the analysis). The integral content of the physicians’ questionnaire (in French) has been previously published [12].

#### Questionnaire for persons living with CP

The telephone questionnaire included nine sociodemographic questions, six questions about pain characteristics, and questions to measure patients’ perceived risks towards medications listed in the MQS-4.0 (above-mentioned 0–10 scales). Similarly to physicians, the meaning of overall risk was explained to the participants, but this time, in accessible language: “*We will go through each of the medications you use for your pain*,* psychological well-being*,* or sleep. For each of these medications*,* I will ask you about your perception of the extent of their side effects. Note that by side effect*,* we mean effects that may bother you in the short or long term*,* such as stomach problems*,* constipation*,* nausea*,* dry mouth*,* decreased libido*,* interactions with other medications*,* dependency*,* abuse*,* insomnia*,* tolerance*,* increased pain over time*,* memory or concentration problems”*. They were instructed to fetch their list of medications or pill containers before the interview. Unlike physicians, persons living with CP were only asked to rate their perceptions towards medications recently used (currently or in the last three months prior to the interview). They were instructed to fetch their list of medications or pill containers before the interview The questionnaire was pretested with three patient partners. The integral content of the patients’ questionnaire (in French) is presented in Additional file 2.

### Statistical analysis

We used descriptive statistics for participants’ sociodemographic profiles (means and standard deviations for continuous variables; numbers and proportions for categorical variables). Perceived risks by physicians and persons living with CP were described (means and standard deviations), and differences between the two groups were assessed using Mann-Whitney U tests. To better capture clinically important differences, we focused our results section on medication subclasses for which the differences in mean risk scores were greater than 2 points and statistically significant (*p* < 0.05). The arbitrary cutoff point of 2-points was used to ease the interpretation of our data. Differences interpretation and bivariate tests were not applied when the number of persons living with CP using a specific medication subclass was too small (13 out of 36 subclasses had ≤ 6 persons using them). As web-based recruitment methods tend to oversample women (women are more likely to engage in online environments and use social media more frequently [22, 23]), the main results were stratified by gender identity (men and women) to assess the presence of sample bias.

## Results

We recruited 83 physicians in this study (see Table 1). The majority identified as women (89.2%), were practising in various regions across Quebec (all 17 administrative regions were represented), and were practising in primary care clinics (44.6%). The majority (51.6%) had over 10 years of experience, and 21.7% self-identified as specialists in CP treatment. A total of 141 patients were recruited for the study. The average age of the patients was 54.5 years (± 11.6), and they had been living with CP for an average of 18.5 years (± 13.6) (see Table 2). On a scale of 0 to 10, patients rated their pain over the last 7 days at an average of 5.8 (± 2.1). Most of the sample identified as women (85.8%); they resided in almost all regions of Quebec. The main diagnoses (self-reported as established by a physician or nurse practitioner and classified by our team according to the International Classification of Diseases 11th Revision [1]) were: chronic widespread pain (44.7%), osteoarthritis (30.5%), chronic neuropathic pain (11.3%), herniated disc (7.8%), chronic migraine (5.0%), and chronic post-traumatic pain (4.3%) (non mutually exclusive categories).

**Table 1:** Physicians’ sociodemographic data

Variables	Physicians (*n* = 83) *n* (%)
Sex at birth*	
Females	75 (90.36)
Males	8 (9.64)
Gender Identity*	
Women	74 (89.16)
Men Other	9 (10.84) 0 (0.00)
**Region of residence**† Nonremote regions Remote regions	72 (86.75) 11 (13.25)
**Years in practice**	
0–5	23 (27.71)
6–10 11–20 21 and +	18 (21.67 32 (38.55) 10 (12.05)
**Type of practice**	
Primary care clinic Hospital setting Pain clinic Emergency room Local community services centre (CLSC) Other ‡	37 (44.58) 22 (26.51) 9 (10.84) 5 (6.02) 4 (4.82) 6 (7.23)
**Self-identification as a pain treatment specialist**	
Yes	18 (21.69)
No	65 (78.31)

Footnotes:

No missing data

* Gender identity (social construct) differed from sex at birth (biological attributes) for 1.2% of participants

† Revenu Quebec defines remote resource regions as: Bas-Saint-Laurent (region 01), Saguenay–Lac-Saint-Jean (region 02), Abitibi-Témiscamingue (region 08), Côte-Nord (region 09), Nord-du-Québec (region 10), Gaspésie–Îles-de-la-Madeleine (region 11). Nonremote regions are near a major urban centre

‡ Other settings include outpatient clinic, rehabilitation center, operating room, palliative care

**Table 2:** Patients’ sociodemographic data

Variables	Patients (*n* = 141)
	Mean ± SD
**Age (y)**	54.47 ± 11.56
**Duration of pain (y)**	18.49 ± 13.64
**Pain intensity in the last 7 days (0–10)**	5.80 ± 2.13
	n (%)
Sex at birth* Females Males	120 (85.71) 20 (14.29)
**Gender Identity**	
Women	121 (85.82)
Men	20 (14.18)
Other	0 (0.00)
**Region of residence**†	
Nonremote regions	111 (78.72)
Remote regions	30 (21.28)
**Report of having received a diagnostic by a physician or nurse practitioner (non mutually exclusive categories)**	
Chronic widespread pain	63 (44.68)
Osteoarthritis (and arthrosis)	43 (30.50)
Chronic neuropathic pain	16 (11.35)
Herniated disc	11 (7.80)
Chronic migraine	7 (4.96)
Chronic post traumatic pain	6 (4.26)
Not diagnosed	14 (9.93)

Footnotes:

* 1 missing data

† Revenu Quebec (provincial revenue agency) defines remote resource regions as: Bas-Saint-Laurent (region 01), Saguenay–Lac-Saint-Jean (region 02), Abitibi-Témiscamingue (region 08), Côte-Nord (region 09), Nord-du-Québec (region 10), Gaspésie–Îles-de-la-Madeleine (region 11). Nonremote regions are near a major urban centre

SD = Standard Deviation

Table 3 describes the perceived risks for physicians and persons living with CP regarding 36 assessed medication subclasses. Among physicians, the highest perceived risk scores (average ≥ 6/10) were for: acetaminophen in combination with an opioid (6.2/10), short-acting opioids (7.9/10), long-acting opioids (7.2/10), opioids associated with a norepinephrine reuptake inhibition (e.g., tramadol) (6.2/10), barbiturates (7.6/10), benzodiazepines (7.5/10) and oral corticosteroids (6.7/10). For persons living with CP, only two subclasses had ratings ≥ 6/10, and they were entirely different subclasses: partial opioid receptor agonists (e.g., buprenorphine) (6/10) and clonidine (7.3/10). Among both physicians and patients, antidepressants did not emerge as one of the most concerning subclasses (average < 4.6/10). Both physicians and patients perceived the risks of using acetaminophen as quite low (2.3/10 and 1.2/10, respectively).

**Table 3:** Perceived risks for physicians and persons living with chronic pain

Medication subclasses	Physicians		Persons living with CP		Difference		*p*-value*
	N	Mean ± SD (0–10 scores)	N	Mean ± SD (0–10 scores)			
NSAIDs – selective cyclooxygenase 2 inhibitors (COX-2)	83	4.71 ± 1.94	23	3.30 ± 3.42	1.41		**0.035**
NSAIDs – salicylates	83	5.22 ± 2.01	1	-	NA		NA
Other oral NSAIDs (non-specific)	80	5.45 ± 1.73	58	2.91 ± 3.38	2.54		**≤ 0.001**
NSAIDs – Topical agents	83	1.94 ± 1.46	38	0.45 ± 1.64	1.49		**≤ 0.001**
Various topical agents	83	1.98 ± 1.51	31	0.77 ± 1.84	1.21		**≤ 0.001**
Acetaminophen	83	2.27 ± 1.57	98	1.15 ± 2.51	1.12		**≤ 0.001**
Acetaminophen in combination with an opioid	82	6.22 ± 1.71	14	3.57 ± 3.76	2.65		**0.017**
Short-acting opioids	82	7.93 ± 1.46	36	5.81 ± 3.74	2.12		**0.021**
Long-acting opioids	83	7.20 ± 1.51	13	3.62 ± 3.43	3.58		**≤ 0.001**
Opioids associated with a norepinephrine reuptake inhibition (e.g., tramadol)	82	6.20 ± 1.84	26	4.35 ± 3.62	1.85		**0.015**
Partial opioid receptor agonists	74	5.81 ± 1.73	5	6.00 ± 2.74	NA		NA
Opioids in combination with an opioid receptor antagonist	77	5.91 ± 1.67	6	4.67 ± 4.55	NA		NA
Anticonvulsants – Calcium channel blockers (gabapentinoids)	82	4.85 ± 1.56	53	4.43 ± 3.45	0.42		0.627
Anticonvulsants – Sodium channel blockers	81	5.74 ± 1.45	3	2.67 ± 4.62	NA		NA
Anticonvulsants – Other	80	5.41 ± 1.56	10	4.20 ± 3.82	1.21		0.637
Antidepressants – SNRIs	82	3.87 ± 1.42	56	3.39 ± 3.27	0.48		0.228
Antidepressants – SSRIs	82	3.68 ± 1.51	21	2.86 ± 3.58	0.82		**0.047**
Antidepressants – SSRIs and 5-HT2 receptor antagonists	82	3.62 ± 1.54	15	3.67 ± 3.44	**-**0.05		0.492
Antidepressants – Specific noradrenergic and serotonergic	80	3.99 ± 1.60	3	3.33 ± 4.16	NA		NA
Antidepressants – Tricyclics	80	4.63 ± 1.75	25	2.36 ± 3.12	2.27		**≤ 0.001**
Antidepressants – Miscellaneous	78	3.83 ± 1.40	15	2.53 ± 2.88	1.30		**0.023**
Antipsychotics	79	5.51 ± 1.41	17	2.18 ± 3.00	3.33		**≤ 0.001**
Barbiturates	73	7.58 ± 1.54	0	-	NA		NA
Benzodiazepines	82	7.54 ± 1.48	32	2.50 ± 3.12	5.04		**≤ 0.001**
Various anxiolytics, sedatives, and hypnotics	79	5.76 ± 1.85	13	3.77 ± 3.35	1.99		0.065
Centrally acting skeletal muscle relaxants	81	4.56 ± 1.82	34	4.18 ± 3.14	0.38		0.651
GABA-Derivative skeletal muscle relaxants	76	4.41 ± 1.52	6	4.00 ± 3.63	NA		NA
Miscellaneous muscle relaxants	72	3.94 ± 1.77	2	5.00 ± 2.83	NA		NA
Synthetic cannabinoids (under prescription)	78	5.36 ± 1.76	23	4.26 ± 3.26	1.1		0.087
Medical/therapeutic cannabis	77	5.87 ± 2.10	43	3.09 ± 3.28	2.78		**≤ 0.001**
Antimigraine agents – 5HT-1 receptor agonists (triptans)	79	3.56 ± 1.51	17	3.12 ± 2.85	0.44		0.074
Antimigraine agents – CGRP Antagonists	74	4.04 ± 1.73	0	-	NA		NA
Antimigraine agents – Miscellaneous	70	4.17 ± 1.74	2	1.00 ± 1.41	NA		NA
Corticosteroids – Oral	81	6.72 ± 1.85	3	3.33 ± 3.51	NA		NA
Clonidine	78	4.24 ± 1.81	3	7.33 ± 2.52	NA		NA
Mexiletine	67	5.54 ± 2.06	0	-	NA		NA

Footnotes:

*Mann-Whitney U tests; Bold values denote statistical significance at the p<0.05 level

CP = Chronic pain; NSAIDS = Non-Steroidal Anti-Inflammatory Drugs; COX-2 = selective cyclooxygenase 2 inhibitors; SNRIs = Serotonin norepinephrine reuptake inhibitors; SSRIs = Selective serotonin reuptake inhibitors; CGRP = Calcitonin gene-related peptide. NA = Non applicable, i.e., the number of persons living with CP who were using that specific medication subclass was too small for statistical testing/valid estimation of difference

There were eight subclasses where the difference in mean risk score between physicians and patients was > 2 points and statistically significant (*p* < 0.05): non-specific oral NSAIDs, acetaminophen in combination with an opioid, short-acting opioids, long-acting opioids, tricyclic antidepressants, antipsychotics, benzodiazepines, and medical cannabis. For these subclasses, physicians consistently perceived higher risks than persons living with CP. The same trends were observed for all those medication subclasses when the analysis was repeated in women and in men (gender identity subgroups), suggesting that sample bias did not affect the quality of our results. The only exception was among men physicians and patients, where the mean perceived risks associated with short-acting opioids were similar. In the entire cohort, patients did not, in any case, report a statistically significant higher perception of risks than physicians for any of the medication subclasses.

## Discussion

This study aimed to compare the perceptions of physicians and persons living with CP regarding the risks of adverse drug reactions associated with different medication subclasses used for pain and associated comorbidity treatment. The findings revealed a consistent trend where physicians perceived higher risks than persons living with CP for several commonly used medication subclasses, including non-specific NSAIDs, acetaminophen in combination with an opioid, short-acting opioids, long-acting opioids, tricyclic antidepressants, antipsychotics, benzodiazepines, and medical cannabis.

Most persons living with CP (62–93%) [24–27] use medications and co-medications for pain management, with a significant proportion using both prescribed and over-the-counter medications. In the province of Quebec, where the present study was conducted, it was estimated that 29% used prescribed medications exclusively, 15% used over-the-counter medications exclusively, and 56% used both [27]. This suggests that self-medication is present and can contribute to the risk [28]. It should be pointed out that although pharmacological interventions play a role in CP management, they are generally not considered first-line treatment options. Multimodal approaches prioritizing non-pharmacological strategies, such as physical therapy, psychological interventions, lifestyle modifications, and self-management should be emphasized [5]. Raising awareness and educating patients about the risks associated with pain medication use remain crucial, whether the medications are prescribed or obtained over-the-counter, to ensure safer and more effective management of CP.

Based on our results, prescribed medications as well as medications available over-the-counter should receive special attention from physicians, pharmacists and nurse practitioners to ensure that patients are well informed about the risks and benefits of their treatment options. This is particularly relevant since our results showed that patients tended to underestimate the risks associated with medication compared to physicians. This underscores the importance of facilitating a more extensive discussion on medication risks to empower patients to make informed decisions and participate in shared decision-making regarding their treatments. Such discussions can also enable prescribers to adopt a more personalized approach, tailoring prescriptions to the individual patient’s risk-benefit profile. While clinical practice guidelines remain a crucial resource for guiding treatment decisions, standardized tools, like the MQS-4.0 medication list [12], could be used in clinical practice to facilitate these discussions (standardized list that can be used as a tool), along with the development of educational interventions for persons living with CP.

In our study, some medication risk scores given by physicians and patients were quite low, particularly for acetaminophen (2.3/10 for physicians and 1.2/10 for patients). Despite its perceived safety for many medical conditions, acetaminophen’s hepatotoxicity makes it a leading cause of liver transplants [29, 30]. This highlights the need to consider the risks-benefit ratio of acetaminophen use in the context of CP, where it is often taken daily for extended periods, versus in the context of acute pain [31]. It was also surprising that antidepressants did not emerge among the most concerning subclasses in the eyes of our participants, considering their numerous adverse drug reactions (e.g., serotonin syndrome, weight gain, sexual dysfunction) [32]. Given the complexity and risks of opioid pharmacotherapy, future studies could benefit from categorizing opioids into weak (e.g., codeine, tramadol) and potent (e.g., fentanyl, morphine sulphate, oxycodone, buprenorphine) forms for more meaningful clinical comparisons. Exploring differences in risk perception based on opioid potency would provide valuable insights for tailoring clinical practice and education.

While this study highlights the need for enhanced patient education regarding medication risks, the role of healthcare providers in this process must also be considered. Physicians and other healthcare professionals have significant responsibility for ensuring that patients and caregivers receive clear and evidence-based information about medications’ risks and benefits [33, 34]. Shared decision making can be enhanced using different means (e.g., decision aids and question prompt lists [33]). Recently, Resnicow et al. [34] have suggested that shared decision making could be patient-driven or provider-driven depending on clinical factors or the patient’s personal characteristics. However, many studies highlight the challenges physicians face in accurately assessing treatment’ risks and benefits (Hoffman et al., 2017; Morgan et al., 2021). A systematic review by Hoffmann et al. highlighted that clinicians often held inaccurate expectations regarding the benefits and harms. Educating physicians on risk communication techniques and shared decision-making models (Bomhof-Roordink et al., 2019) could strengthen this process and improve treatment outcomes. Future interventions could focus on both patient and healthcare providers education to ensure informed, collaborative, and effective chronic pain management.

Further research, including qualitative inquiries, could provide deeper insights into the factors influencing patients’ perception of pain medications and co-medications risks. The methodological choices we have made partly explain the differences we observed between physicians and patients. For example, telephone conversations with patients have allowed us to understand that for some patients, the risk of a medication was perceived as the frequency of occurrence of an adverse drug reaction, while for others, it was the functional impact of the medication. A patient’s perception of risks may be influenced by their knowledge, beliefs, experiences with medication, cultural background, relationship with healthcare professionals, and disease-related factors (e.g., general health and pain tolerance) [35–37]. Stratification was conducted based on gender identity, because our sample contained a higher proportion of women than probabilistic samples of people living with chronic pain. Our stratified analysis by gender identity did not reveal differences in risk perceptions. This suggests that the over-representation of women did not lead to an over or underestimation of risk perception. However, as the subsample of persons identifying as men was small, new studies should further explore gender identity differences in terms of pain medication risk perceptions.

### Limitations

This study has limitations. First, the use of a 0–10 numerical scale to assess perceived risks introduces subjectivity, as participants may interpret “high risk” differently based on personal experiences, knowledge, or tolerance levels. Without a standardized reference or baseline, the data may reflect individual attitudes rather than an objective measure of risk perception. Additionally, the context of adverse drug reactions (e.g., severity, likelihood) was not explicitly incorporated into the assessment. Minor adverse drug reactions may be perceived as acceptable even at higher frequencies, while even small risks of life-threatening outcomes could be intolerable. This imperfect contextualization represents a limit. Second, physicians and patients assessed different aspects of risk: physicians rated risks for the general population and based on evidence, while patients rated risks based on personal adverse drug reaction experienced and their own knowledge. This misalignment may decrease the meaning of comparisons, but still, the observed differences highlight gaps that can inform patient-physician communication and shared decision-making. Furthermore, the low number of chronic pain specialists among participating physicians may have influenced the risk ratings, as non-specialists might have less specific knowledge in chronic pain management. Nevertheless, our sample was representative of the physician workforce in the province of Quebec, including a majority of family physicians and specialties not focused on pain management [38]. Third, the study focused solely on perceived risks without evaluating perceived effectiveness. Decision-making about medication often involves weighing the benefits against the risks, and this omission leaves an incomplete understanding of how participants make these trade-offs. Future research should integrate assessments of both risk and effectiveness to provide a more comprehensive perspective on decision-making in chronic pain management. Finally, for certain subclasses of medication relevant for CP treatment (e.g., partial opioid receptor agonists, opioids associated with an opioid receptor antagonist, antimigraine agents, oral corticosteroids), too few patients living with CP in our sample were using them. This may have affected the accuracy of our estimates and our ability to statistically compare the risk perceptions of patients and physicians. Everything suggests that this reflects a low prevalence of use in the community. However, we believe that this does not affect our ability to draw valid conclusions regarding the most used classes (e.g., non-specific NSAIDs, acetaminophen, gabapentinoids, anticonvulsants, SNRIs). We must also consider that we conducted the study in a population of prevalent medication users, who likely tolerate their medication well. However, their perceptions still allowed us to identify subclasses of medications that differed more from physicians’ perceptions and still enabled us to identify education targets.

## Conclusions

The physician-patient partnership is central to ensuring quality of care and safe medication use [39]. The substantial differences observed between physicians’ perceptions and those of persons living with CP demonstrate the importance of continuing efforts to educate patients about their condition and their medication, particularly in the context of prolonged use for CP. This education is important to promote treatment adherence and reduce medication misuse and risks, especially for specific medication subclasses.

## Electronic supplementary material

Below is the link to the electronic supplementary material.

Supplementary Material 1

Supplementary Material 2

## Data Availability

The datasets are not publicly available because participants did not initially provide consent for open data sharing. However, the data is available from the corresponding author upon reasonable request and conditionally to proper ethical approval for anonymous secondary data analysis.
