# Supplementary material for: Physicians’ and patients’ perceived risks of chronic pain medication and co-medications in Quebec, Canada: a cross-sectional study
Source: BMC Prim Care. 2025 Jan 14;26:8. doi: 10.1186/s12875-025-02704-5 (PMC11730153; doi:10.1186/s12875-025-02704-5)
Supplement: Supplementary file 1 — Supplementary Material 1 [file 12875_2025_2704_MOESM1_ESM.pdf]

Additional file 1

The items of the MSQ-4.0, including examples of various medications in each subclass.

MEDICATION SUBCLASS	EXAMPLE OF MEDICATIONS
NSAIDs – selective cyclooxygenase 2 inhibitors (COX-2)	celecoxib (Celebrex®)
NSAIDs – salicylates	acetylsalicylic acid (ASA, Aspirin®)
Other oral NSAIDs	ibuprofen (Advil®, Motrin®), naproxen (Naprosyn®), diclofenac, ketoprofen, meloxicam, piroxicam
NSAIDs – Topical agents	diclofenac sodium (Pennsaid®), diclofenac diethylamine (Voltaren Emulgel®)
Various topical agents	various magistral preparations, ketamine cream, benzocaine, lidocaine (Emla®), capsaicin (Zostrix®), Antiphlogistine®, Myoflex®, Tiger Balm®
Acetaminophen	Tylenol®
Acetaminophen in combination with an opioid	acetaminophen with codeine (Triatec-30®, Empracet®), with tramadol (Tramacet®) or with oxycodone (Percocet®)
Short-acting opioids	codeine, fentanyl, hydromorphone, morphine, oxycodone
Long-acting opioids	methadone, extended release formulations (Codeine Contin® OxyContin®, Hydromorph Contin®, Jurnista®, MS Contin®, Duragesic®)
Opioids associated with norepinephrine reuptake inhibition	tramadol, tapentadol
Partial opioid receptor agonists	buprenorphine (Butrans®), butorphanol
Opioids in combination with an opioid receptor antagonist	buprenorphine/naloxone (Suboxone®), oxycodone/naloxone (Targin®)
Anticonvulsants – Calcium channel blockers (gabapentinoids)	pregabalin (Lyrica®), gabapentin (Gabapentin®)
Anticonvulsants – Sodium channel blockers	oxycarbazepine, Lamotrigine (Lamictal®)
Anticonvulsants – Other	Levetiracetam (Keppra®), topiramate (Topamax®)
Antidepressants – Serotonin norepinephrine reuptake inhibitors (SNRIs)	duloxetine (Cymbalta®), venlafaxine (Effexor XR®)
Antidepressants – Selective serotonin reuptake inhibitors (SSRIs)	citalopram (Celexa®), escitalopram, fluvoxamine (Luvox®), fluoxetine (Prozac®), paroxetine (Paxil®), sertraline (Zoloft®)
Antidepressants – Serotonin reuptake inhibitors and 5-HT2 receptor antagonists	trazodone
Antidepressants – Specific noradrenergic and serotonergic	mirtazapine (Remeron®)
Antidepressants – Tricyclic	amitriptyline (Elavil®), nortriptyline (Aventyl®), desipramine
Antidepressants – Miscellaneous	bupropion (Wellbutrin®)

Antipsychotics	aripiprazole, chlorpromazine, clozapine, haloperidol, olanzapine (Zyprexa®), quetiapine (Seroquel®)
Barbiturates	phenobarbital, primidone
Benzodiazepines	lobazam, clonazepam (Rivotril®), alprazolam (Xanax®), bromazepam, chlordiazepoxide, diazepam (Valium®), flurazepam, lorazepam (Ativan®), midazolam, oxazepam
Various anxiolytics, sedatives, and hypnotics	buspirone, hydroxyzine (Atarax®), promethazine, zopiclone, zolpidem tartrate
Centrally acting skeletal muscle relaxants	cyclobenzaprine (Flexeril®), methocarbamol (Robax®)
GABA-Derivative skeletal muscle relaxants	baclofen (Lioresal®)
Miscellaneous muscle relaxants	orphenadrine (Norflex®)
Synthetic cannabinoid (under prescription)	nabilone (Cesamet®)
Medical/therapeutic cannabis	vaporized, vaped, oral, oromucosal, topical
Antimigraine agents – 5HT-1 receptor agonists (triptans)	sumatriptan (Imitrex®)
Antimigraine agents – Calcitonin gene-related peptide (CGRP) Antagonists	erenumab (Aimovig®)
Antimigraine agents – Miscellaneous	pizotifen (Sandomigran®)
Corticosteroids – Oral	prednisone, prednisolone
Clonidine (antihypertensive)	
Mexiletine (antiarrythmics)	
