# Supplementary material for: Physicians’ and patients’ perceived risks of chronic pain medication and co-medications in Quebec, Canada: a cross-sectional study
Source: BMC Prim Care. 2025 Jan 14;26:8. doi: 10.1186/s12875-025-02704-5 (PMC11730153; doi:10.1186/s12875-025-02704-5)
Supplement: Supplementary file 2 — Supplementary Material 2 [file 12875_2025_2704_MOESM2_ESM.pdf]

UQAT
LABORATOIRE DE RECHERCHE
EN ÉPIDÉMIOLOGIE DE LA
DOULEUR CHRONIQUE

Questionnaire téléphonique pour les patients - MQS 4.0

Mot d'introduction et consentement

Présentation téléphonique

Se présenter / Remise en contexte suite au courriel

Valider si la personne a bien lu le formulaire de consentement (réponse courriel bien reçu de notre part) et répondre à ses questions

Demander au patient si c’est un bon moment pour faire l’entrevue. Prévoir environ 20 minutes.

Il n'y a aucune question obligatoire, sentez-vous à l'aise de ne pas répondre à certaines questions si elles vous rendent inconfortables

* 1. **Confirmation des critères d'inclusion**

- ☐ Vous sentez-vous apte à et libre de participer au présent projet de recherche?
- ☐ Confirmez-vous avoir plus de 18 ans?
- ☐ Confirmez-vous avoir de la douleur de façon constante ou occasionnelle depuis plus de 3 mois (peu importe si cette douleur est légère ou sévère, peu importe l’endroit, et peu importe la cause)?
- ☐ Avez-vous utilisé des médicaments pour le traitement de votre douleur dans la dernière année?

2. Identifiant (initiales)

☐

LABORATOIRE DE RECHERCHE
EN ÉPIDÉMIOLOGIE DE LA
DOULEUR CHRONIQUE

Questionnaire téléphonique pour les patients - MQS 4.0

Caractéristiques des participants

3. **Quel âge avez-vous (18-120)**

4. **Quel était votre sexe à la naissance?**

- ◯ Féminin
- ◯ Masculin

5. **Sélectionnez l'option qui correspond le mieux à votre identité de genre actuelle**

- ◯ Genre fluide
- ◯ Homme
- ◯ Femme
- ◯ Non binaire
- ◯ Homme trans
- ◯ Femme trans
- ◯ Bispirituel / Bispirituelle
- ◯ Je ne m'identifie à aucune option proposée

6. **Dans quelle région du Québec résidez-vous?**

- ◯ Abitibi-Témiscamingue
- ◯ Bas-Saint Laurent
- ◯ Capitale Nationale
- ◯ Centre-du-Québec
- ◯ Chaudière-Appalaches
- ◯ Côtes-Nord
- ◯ Estrie
- ◯ Gaspésie-Îles-de-la-Madeleine
- ◯ Lanaudière
- ◯ Laurentides
- ◯ Laval
- ◯ Mauricie
- ◯ Montérégie
- ◯ Montréal
- ◯ Nord-du-Québec
- ◯ Outaouais
- ◯ Saguenay-Lac-Saint-Jean
- ◯ Autre (veuillez préciser)

  ____

- ◯ Je pratique dans une autre province canadienne

7. **Quel est votre pays de naissance?**

- ◯ Canada
- ◯ Autre

8. **Êtes-vous autochtone, c’est-à-dire, Première Nation, Métis ou Inuk (Inuit)?**

- ◯ Non
- ◯ Oui, Première Nation
- ◯ Oui, Métis
- ◯ Oui, Inuk (Inuit)

9. **Niveau de scolarité** (sélectionnez le plus haut niveau atteint)

- ◯ Sans diplôme d’études secondaires
- ◯ Diplôme d'études secondaires ou l'équivalent
- ◯ Certificat ou diplôme d'apprenti inscrit, d'une école de métiers ou d'un centre de formation professionnelle? (ex. coiffure, cuisine, électricien, charpenterie)
- ◯ Certificat ou diplôme d'études collégiales, d'un CÉGEP ou d'un autre établissement non universitaire (ex. technique de comptabilité, technique de génie industriel, adjoint juridique, DEC préuniversitaire, etc.)
- ◯ Programme universitaire de 1er cycle (ex. baccalauréat, certificat)
- ◯ Programme universitaire de 2e cycle (ex. maîtrise, D.E.S.S., microprogramme de 2e cycle)
- ◯ Doctorat (ex. MD, PharmD, Ph.D., D.Psy., D.Ed.)

10. **Quel statut d’emploi représente le mieux votre situation?**

- ◯ Travaille présentement à temps plein
- ◯ Travaille présentement à temps partiel
- ◯ À la recherche d'emploi / sans emploi
- ◯ Congé de maladie
- ◯ Congé de maternité
- ◯ Invalidité temporaire ou permanente en raison de votre douleur
- ◯ Invalidité pour d’autres raisons que la douleur
- ◯ Étudiant
- ◯ Mise à pied temporaire (chômage planifié)
- ◯ Retraité(e)
- ◯ Au foyer
- ◯ Inconnu
- ◯ Autre (veuillez préciser)

11. **En général, diriez-vous que votre santé est :**

- ◯ Excellente
- ◯ Très bonne
- ◯ Bonne
- ◯ Passable
- ◯ Mauvaise

LABORATOIRE DE RECHERCHE
EN ÉPIDÉMIOLOGIE DE LA
DOULEUR CHRONIQUE

Questionnaire téléphonique pour les patients - MQS 4.0

Votre perception du risque lié aux médicaments utilisés pour le traitement de la douleur chronique

Demander au patient d'aller chercher sa liste de médicaments ou encore ses boitiers. Lui expliquer les consignes et les définitions et lui demander de les énumérer.

Pour chacun d'eux, évaluer la perception du risque (répéter l'échelle chaque fois**).**

Consignes à lire au répondant :
Nous passerons un à un les médicaments que vous utilisez pour votre douleur, votre bien être psychologique ou votre sommeil. Pour chacun de ces médicaments, je vous demanderai votre perception de l'ampleur de leurs effets secondaires.

À noter que nous entendons par effets secondaires des effets qui peuvent vous déranger à court ou long terme comme par exemple des problèmes d'estomac, constipation, nausées, bouche sèche, baisse de libido, interactions avec d'autres médicaments, dépendance, abus, insomnie, tolérance, augmentation de la douleur au fil du temps, problèmes de mémoire ou de concentration.

0 signifie aucun effet secondaire et 10, des effets secondaires très dérangeants au quotidien

À la fin, vérifier que la personne n'a rien oublié si rien n'est coché dans certaines sections (ex. « Est-ce qu'il reste d'autres de vos médicaments que nous n'avons pas abordés? Tylenol, Advil, Robax, Antidépresseur, Cannabis pour la douleur?)

12. **Veuillez choisir sur l'échelle suivante ce qui représente le mieux votre perception du risque associé à l'utilisation de ces médicaments.**

À noter que nous entendons par "risque" le potentiel d'amener un effet indésirable à court ou long terme. En voici des exemples : problèmes d'estomac, constipation, nausées, bouche sèche, baisse de libido, interactions avec d'autres médicaments, dépendance, abus, insomnie, tolérance, augmentation de la douleur au fil du temps, problèmes de mémoire ou de concentration.

	0 = Aucun risque et 10 = Risque très élevé
**AINS inhibiteurs sélectifs de la cyclooxygénase 2 (COX-2)**<br>Ex. célécoxib (Celebrex®)	

	0 = Aucun risque et 10 = Risque très élevé
**AINS - salicylates**<br>Ex. acide acétylsalicylique (AAS, Aspirine®)	
**Autres AINS oraux**<br>Ex. ibuprofène (Advil®, Motrin®), naproxène (Naprosyn®), diclofénac, kétoprofène, méloxicam, piroxicam	
**AINS - Topiques**<br>Ex. diclofenac sodique (Pennsaid®), diclofenac diéthylamine (Voltaren Emulgel®)	
**Agents topiques divers**<br>Ex. préparations magistrales diverses, crème de kétamine, benzocaïne, lidocaïne (Emla®), capsaïcine (Zostrix®), Antiphlogistine®, Myoflex®, Tiger Baulm®	
**Acétaminophène**<br>Ex. Tylenol®	
**Acétaminophène en combinaison avec un opioïde**<br>Ex. acétaminophène avec codéine (Triatec-30®, Empracet®), avec tramadol (Tramacet®) ou avec oxycodone (Percocet®)	
**Opioïdes à courte action**<br>Ex. codéine, fentanyl, hydromorphone, morphine, oxycodone	
**Opioïdes à longue action**<br>Ex. méthadone, formules à libération prolongée (Codeine Contin® OxyContin®, Hydromorph Contin®, Jurnista®, MS Contin®, Duragesic®)	
**Opioïdes associés à un effet IRN**<br>Ex. tramadol, tapentadol	

0 = Aucun risque et 10 = Risque très élevé

**Agonistes partiels des récepteurs opiacés** Ex. buprénorphine (Butrans®), butorphanol, pentazocine (Talwin®)	
**Opioïdes en combinaison avec un antagoniste des récepteurs opiacés** Ex. buprenorphine/naloxone (Suboxone®), oxycodone/naloxone (Targin®)	
**Anticonvulsivants – Bloqueurs des canaux calciques (gabapentinoïdes)** Ex. prégabaline (Lyrica®), gabapentine (Gabapentin®), Neurontin®	
**Anticonvulsivants – Bloqueurs des canaux sodiques** Ex: oxycarbazépine, Lamotrigine (Lamictal®), phénytoïne (Dilantin®), carbamazépine (Tégrétol®)	
**Autres anticonvulsivants** Ex. Lévétiracétam (Keppra®), topiramate (Topamax®)	
**Antidépresseurs - Inhibiteurs sélectifs de la recapture de la sérotonine et de la norépinéphrine (IRSN)** Ex. duloxetine (Cymbalta®), venlafaxine (Effexor XR®)	
**Antidépresseurs - Inhibiteurs sélectifs de la recapture de la sérotonine (ISRS)** Ex. citalopram (Celexa®), escitalopram, fluvoxamine (Luvox®), fluoxétine (Prozac®), paroxetine (Paxil®), sertraline (Zoloft®)	

	0 = Aucun risque et 10 = Risque très élevé
**Antidépresseurs - Inhibiteurs du recaptage de la sérotonine et antagonistes des récepteurs 5-HT2**<br>Ex. trazodone	
**Antidépresseurs - Noradrénergiques et sérotoninergiques spécifiques**<br>Ex : mirtazapine (Remeron®)	
**Antidépresseurs - Tricycliques**<br>Ex. amitriptyline (Elavil®), nortriptyline (Aventyl®), desipramine	
**Antidépresseurs - Divers**<br>Ex. bupropion (Wellbutrin®)	
**Antipsychotiques**<br>Ex. aripiprazole, chlorpromazine, clozapine, haloperidol, olanzapine (Zyprexa®), quétiapine (Seroquel®)	
**Barbituriques**<br>Phénobarbital, phénobarbital, primidone	
**Benzodiazépines**<br>Ex. clobazam, clonazépam (Rivotril®), alprazolam (Xanax®), bromazépam, chlordiazépoxide, diazépam (Valium®), flurazépam, lorazépam (Ativan®), midazolam, oxazépam	
**Anxiolytiques, sédatifs et hypnotiques divers**<br>Ex. buspirone, hydroxyzine (Atarax®), prométhazine, zopiclone, zolpidem tartrate	
**Myorelaxants à action centrale**<br>Ex. cyclobenzaprine (Flexeril®), méthocarbamol (Robax®)	

	0 = Aucun risque et 10 = Risque très élevé
**Myorelaxants dérivés du GABA** Ex. baclofène (Lioresal®)	
**Myorelaxants divers** Ex. orphénadrine (Norflex®)	
**Cannabinoïdes pharmaceutiques sous prescription** Ex. nabilone (Cesamet®), nabiximols (Sativex®)	
**Cannabis médical/thérapeutique** Ex: vaporisé, vapoté, oral, oromucosal, topique	
**Antimigraineux - Agonistes des récepteurs de 5HT-1 (triptans)** Ex : sumatriptan (Imitrex®)	
**Antimigraineux - Antagonistes du Calcitonin gene-related peptide (CGRP)** Ex. erénumab (Aimovig®)	
**Antimigraineux divers** Ex. pizotifène (Sandomigran®)	
**Corticostéroïdes - Oraux** Ex. prednisone, prednisolone	
**Clonidine** (antihypertenseur)	
**Mexilétine** (antiarythmique)	

13. **Commentaires**

LABORATOIRE DE RECHERCHE EN ÉPIDÉMIOLOGIE DE LA DOULEUR CHRONIQUE

Questionnaire téléphonique pour les patients - MQS 4.0

Caractéristiques de la douleur des participants

14. **Depuis combien de temps ressentez-vous votre douleur?**

En jours: ____

OU En mois: ____

OU En Années ____

15. **Veuillez choisir sur l'échelle de 0 à 10 le chiffre qui décrit le mieux la douleur que vous avez ressentie en moyenne ou en général au cours des 7 derniers jours**

Si vous souffrez de douleur dans plus d'une région du corps, veuillez répondre en tenant compte de l'endroit qui fait le plus mal

0 (aucune douleur) — 10 (la pire douleur possible) ____

Dans quelle(s) régions du corps ressentez-vous de la douleur?

16. **Douleurs généralisées dans tout le corps (ou presque)**

- ○ Oui
- ○ Non

17. Tête

- ○ Oui
- ○ Non

18. Visage

- ○ Oui
- ○ Non

19. Cou

- ◯ Oui
- ◯ Non

20. Épaule(s)

- ◯ Oui
- ◯ Non

21. Bras

- ◯ Oui
- ◯ Non

22. Coude(s)

- ◯ Oui
- ◯ Non

23. Poignet(s)

- ◯ Oui
- ◯ Non

24. Main(s)

- ◯ Oui
- ◯ Non

25. Haut du dos

- ◯ Oui
- ◯ Non

26. Bas du dos

- ◯ Oui
- ◯ Non

27. Poitrine

- ◯ Oui
- ◯ Non

28. Abdomen / Estomac

- Oui
- Non

29. Hanche(s)

- Oui
- Non

30. Fesse(s)

- Oui
- Non

31. Région anale

- Oui
- Non

32. Région génitale

- Oui
- Non

33. Jambe(s)

- Oui
- Non

34. Genou(x)

- Oui
- Non

35. Cheville(s)

- Oui
- Non

36. Pied(s)

- Oui
- Non

37. Autre

38. **Avez-vous reçu un diagnostic par un médecin ou une infirmière praticienne pour votre douleur?**

- Non
- Si oui (veuillez préciser)

39. **Mis à part des médicaments, utilisez-vous actuellement un ou plusieurs traitements non pharmacologiques pour votre douleur (ex. exercice, physiothérapie, psychothérapie, acupuncture, méditation, etc.)?**

- Oui
- Non

Autre (veuillez préciser)

40. **Avez-vous accès à une clinique de douleur?**

- Oui
- Non
- Autre (veuillez préciser)

LABORATOIRE DE RECHERCHE
EN ÉPIDÉMIOLOGIE DE LA
DOULEUR CHRONIQUE

Questionnaire téléphonique pour les patients - MQS 4.0

Fin du questionnaire

Merci beaucoup de votre participation!

Nous sommes très reconnaissants que vous ayez accepté de nous aider dans nos recherches et ce fut un plaisir de parler avec vous aujourd'hui!

41. **Lorsque l'étude sera terminée, souhaiteriez-vous recevoir un résumé des résultats par courrier électronique?**

- Oui
- Non
- Si oui, le consigner

42. **Avez-vous des questions / commentaires?**

UQAT

LABORATOIRE DE RECHERCHE EN ÉPIDÉMIOLOGIE DE LA DOULEUR CHRONIQUE
